# Social defeat-induced anhedonia: effects on operant sucrose-seeking behavior

**DOI:** 10.3389/fnbeh.2015.00195

**Published:** 2015-08-07

**Authors:** Danai Riga, J. Trisna Theijs, Taco J. De Vries, August B. Smit, Sabine Spijker

**Affiliations:** ^1^Department of Molecular and Cellular Neurobiology, Center for Neurogenomics and Cognitive Research, Neuroscience Campus Amsterdam, VU UniversityAmsterdam, Netherlands; ^2^Department of Anatomy and Neurosciences, Neuroscience Campus Amsterdam, VU University Medical CenterAmsterdam, Netherlands

**Keywords:** social defeat-induced persistent stress (SDPS), anhedonia, depression, sucrose self-administration, guanfacine

## Abstract

Reduced capacity to experience pleasure, also known as anhedonia, is a key feature of the depressive state and is associated with poor disease prognosis and treatment outcome. Various behavioral readouts (e.g., reduced sucrose intake) have been employed in animal models of depression as a measure of anhedonia. However, several aspects of anhedonia are poorly represented within the repertoire of current preclinical assessments. We recently adopted the social defeat-induced persistent stress (SDPS) paradigm that models a maintained depressive-like state in the rat, including social withdrawal and deficits in short-term spatial memory. Here we investigated whether SDPS elicited persistent deficits in natural reward evaluation, as part of anhedonia. We examined cue-paired operant sucrose self-administration, enabling us to study acquisition, motivation, extinction, and relapse to sucrose seeking following SDPS. Furthermore, we addressed whether guanfacine, an α_2_-adrenergic agonist that reduces stress-triggered maladaptive behavioral responses to drugs of abuse, could relief from SDPS-induced anhedonia. SDPS, consisting of five social defeat episodes followed by prolonged (≥8 weeks) social isolation, did not affect sucrose consumption during acquisition of self-administration. However, it strongly enhanced the motivational drive to acquire a sucrose reward in progressive ratio training. Moreover, SDPS induced initial resilience to extinction and rendered animals more sensitive to cue-induced reinstatement of sucrose-seeking. Guanfacine treatment attenuated SDPS-induced motivational overdrive and limited reinstatement of sucrose seeking, normalizing behavior to control levels. Together, our data indicate that long after the termination of stress exposure, SDPS induces guanfacine-reversible deficits in evaluation of a natural reward. Importantly, the SDPS-triggered anhedonia reflects many aspects of the human phenotype, including impaired motivation and goal-directed conduct.

## Introduction

One of the most prominent characteristics of depressive disorders is a diminished ability to experience pleasure, a mental state traditionally termed as anhedonia. According to the Diagnostic and Statistical Manual of Mental Disorders (DSM, 5th Edition), anhedonia, or reduced interest in engagement to otherwise rewarding activities, such as socialization and sexual intercourse, is a *key feature* of Major Depressive Disorder (MDD) diagnosis (American Psychiatric Association, 2013). The anhedonic phenotype can occur in presence or in absence of mood-related depressive symptoms, e.g., feelings of sadness or helplessness, and it can persist beyond their recovery (Schrader, 1997). Furthermore, in MDD, anhedonia is strongly associated with predicted severity and persistence of the disorder, as well as poor treatment outcomes (Spijker et al., 2001; Vrieze et al., 2013).

Anhedonia falls in the category of pathologies of the affective domain, and might be largely explained by effects of dysfunctional processing of reinforcing information on mood and cognition. Indeed, depressed individuals suffer cognitive deficits predominantly related to the affective domain (Eshel and Roiser, 2010). In particular in MDD, information processing associated with reward is aberrant, with negative stimuli triggering maladaptive responses, and positive information making only small impact. As a result, depressed patients show defective decision-making and inappropriate behavioral adjustments in face of emotionally charged events (Cella et al., 2010). Conventionally, these deficits are linked to dysfunctional brain circuitry responsible for decision-making, attribution of incentive salience, behavioral reinforcement and expression of motivated behavior (e.g., via the prefrontal cortex and striatum) (Russo and Nestler, 2013).

Emerging revisited concepts of anhedonia (Der-Avakian and Markou, 2012) pose an important question for preclinical models of depression in terms of construct validity, beyond simplistic assessments of consummatory approach, e.g., sucrose intake. Similarly, in light of gaining insight into affected brain circuitry and underlying molecular mechanisms, it is imperative to extend the current anhedonia-parameters' applicability to theories conceptualizing human anhedonia. Previous assessments of the magnitude of anhedonia-like behavior in rodents were based on measurements of preference for a given reward, such as sweetened solutions, without taking into account the contributions of motivational aspects. In particular, sucrose preference-based behavioral readouts fail to dissect reward-related learning, subsequent retention of pleasure-coding information and reward-based decision-making (Ho and Sommers, 2013), unlike operant reward paradigms (Nielsen et al., 2000; Donahue et al., 2014).

Over the years, we adopted an animal paradigm that mimics a sustained depressive-like state in rats, the so-called social defeat-induced persistent stress (SDPS) model. The SDPS paradigm employs an etiologically valid stressor, i.e., social stress in the form of acute social defeat, followed by long-term social isolation (2–3 months) in the absence of chronic sensory interaction with the stressor (Von Frijtag et al., 2000). Social isolation is a necessary component of the SDPS paradigm (De Jong et al., 2005), serving as a sub-threshold stressor that supports the maintenance of the depressive-like state. However, by itself, isolation during adulthood does not induce behavioral or physiological hallmarks of depression (Ruis et al., 1999; Fone and Porkess, 2008; Riga et al., 2014). Using the SDPS model, we and others have established a maintained depressive state in the rat, in which antidepressant-reversible behavioral and neurobiological hallmarks of the human disorder, such as affective and cognitive deficits, reduced neurogenesis, and aberrant physiology of the hippocampus, are present several months after social defeat (Reijmers et al., 2001; Artola et al., 2006; van Bokhoven et al., 2011; Riga et al., 2014). Focusing on such a maintained depressive state, rather than the short-lasting effects of initial stress exposure, has the advantage of modeling the enduring characteristics of human depression. Whereas indicators of acute stress, such as elevated corticosterone levels (van Bokhoven et al., 2011) are absent in this model, in the weeks following social defeat rats gradually develop a sensitization to heterotypic stressors (Buwalda et al., 2005). This is exemplified by increased responsiveness of the HPA axis (Buwalda et al., 1999) and impaired social approach-avoidance lasting up to 6 months (Riga et al., 2014).

With respect to maladaptive processing of reward-associated information, we recently developed a first preclinical model of comorbidity between primary depression and secondary alcohol abuse disorder. SDPS resulted in increased motivation for alcohol and elevated susceptibility to relapse to alcohol-seeking (Riga et al., 2014). Following up on these studies, we questioned here whether the effects of SDPS on reward evaluation are specific to alcohol- and alcohol-signifying cues, or whether they reflect global alterations in the reward-processing system. To this end, we investigated whether SDPS induces an anhedonia-like phenotype by examining its consequences not only regarding consumption, but also with respect to motivation, extinction, and reinstatement toward a non-drug reward, i.e., sweetened water. Furthermore, by employing a pharmacological intervention in the form of a systemic guanfacine administration, previously shown to reverse SDPS-effects on alcohol-seeking (Riga et al., 2014), we aimed to disentangle possible neuronal pathways of depression-induced impairments in motivation and reinforcement.

## Materials and methods

### Animals and social defeat-induced persistent stress (SDPS)

**Figure 1:**
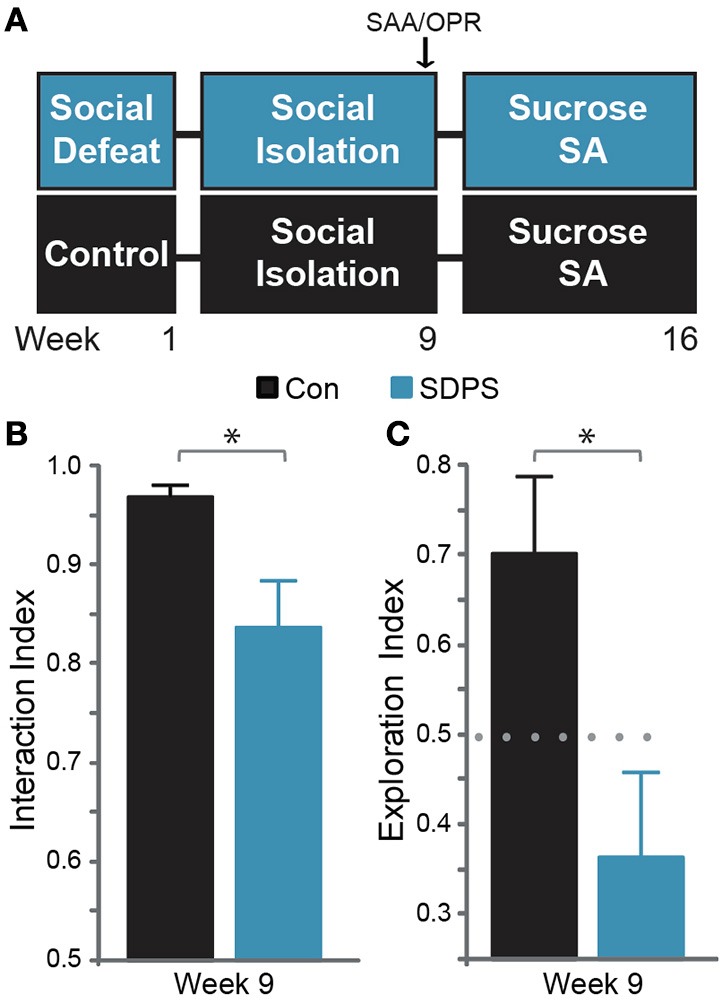
**SDPS induces deficits in the cognitive and the affective domain**. **(A)** SDPS animals were exposed to 5 daily defeat sessions, immediately followed by social isolation (single-housing). Eight weeks following the last defeat episode, Social Approach Avoidance (SAA), and Object Place Recognition tests were employed to assess the effects of SDSP on the affective and cognitive domain. Animals were then subjected to a 7-weeks long operant sucrose self-administration paradigm. **(B)** Whereas both control and SDPS rats spent the majority of time interacting with the unfamiliar social target at the SAA test, SDPS triggered avoidance behavior, indicated by the significantly reduced interaction index as compared to controls. **(C)** SPDS rats failed to retain the position of the displaced object in the short-term spatial memory OPR test, in contrast to controls, which spent the majority of time exploring the object in its new location. ^*^P < 0.050 for Student's *t*-test (gray); dotted gray line indicates the 50% preference index.

Paired-housed male Wistar rats (Harlan CPB, Horst, Netherlands) 6–7 weeks old, weighing < 200 g upon arrival were habituated to the facility (2 weeks), and then were exposed to SDPS (Riga et al., 2014) followed by operant sucrose self-administration (SA) (Figure 1A). During the SDPS paradigm, residents (male Long-Evans, Charles River, UK) were paired-housed with age-matched tube-ligated females (Wistar, Harlan) in order to promote territorial behavior and aggression. The female Wistar and all cage enrichment were removed from the residents' cage before defeat commenced. During the SDPS paradigm, Wistar rats (*n* = 8) were exposed to five daily defeat sessions with the resident, according to the resident-intruder protocol. During the pre- and post-phases of each defeat session (5 min each), the Wistar rat was positioned in the Long Evans home-cage, however, the resident had no access to the intruder due to placement of a transparent, perforated plastic partition-wall. The fight-phase (5 min) was initiated and terminated by respectively, removing or replacing the partition wall. For each defeat session, intruders were matched to a different resident. At defeat days, control animals (*n* = 7) were transferred to the residents' holding room and allowed to explore an empty defeat cage for 15 min. From the first defeat session/cage exposure onwards, all animals were single-housed and remained in social isolation for the rest of the experimental conditions, in absence of further sensory interaction with the stressor (residents). All animals were housed in humidity/temperature-controlled rooms (50%/21 ± 1°C). Food and water were available *ad libitum* for the whole experimental period. All experimental manipulations were conducted during the dark phase of a reversed 12-h light-dark cycle (lights on at 19.00 h). All experiments were approved by the VU University Amsterdam Animal Users Care Committee.

### Assessment of depressive symptomatology

Before participation in any behavioral measurement, all animals were transferred to the video-recording room and habituated to the test arena (plastic, opaque, 79 × 57 × 42 cm) for at least 10 min during 3 consecutive days. Animals were subjected to the Social Approach-Avoidance (SAA) and the Object Place Recognition (OPR) tasks 8 weeks following the last defeat session (Figure 1A).

#### Social approach-avoidance (SAA)

SDPS-induced deficits in social behavior were determined by the SAA test, using an unfamiliar Long-Evans adult male rat (resident) (as adopted by Golden et al., 2011). During test day, Wistar rats were habituated to the testing arena (5 min). A sample phase followed, in which two empty target boxes (TBs, perforated metal, 16 × 7 × 8 cm) were placed on opposite arena walls, and general activity and explorative behavior was measured (5 min). The sample phase was immediately followed by the test phase, in which an unfamiliar resident was introduced to one TB; Wistar rats were then allowed to freely explore and approach either of the TBs (5 min). Approach-avoidance behavior (interaction index) was calculated as time spent in active zone (resident zone)/total exploration time (resident + neutral zone). Active and inactive zones were randomly assigned, to avoid development of preference.

#### Object place recognition (OPR)

Hippocampal-dependent short-term memory was determined with an object place recognition test (Dere et al., 2007) using a 15-min retention interval. Animals were habituated to the test arena as described above. During the sample phase, two identical objects (cylinders or cubes, metal, 8 × 8 × 35 cm) were placed in two opposite corners of the arena, and animals were allowed to explore (5 min). In the test-phase, the previously presented objects were replaced with two identical ones, and one of the objects was displaced to a different corner. Discrimination between the spatial locations of the two objects was used as measurement for spatial memory [exploration index = time spent in novel location/total exploration time (novel + familiar location)] in a 4-min test. The position of novel and familiar locations and object shapes were randomly assigned to avoid development of preference.

All video recordings were analyzed with Viewer^2^ software (BiObserve GmbH, Bonn, Germany). Approach/avoidance behavior (SAA) and retention of spatial memory (OPR) were based on animals' performance at the first minute of each test.

### Cue-paired operant sucrose self-administration

**Figure 2:**
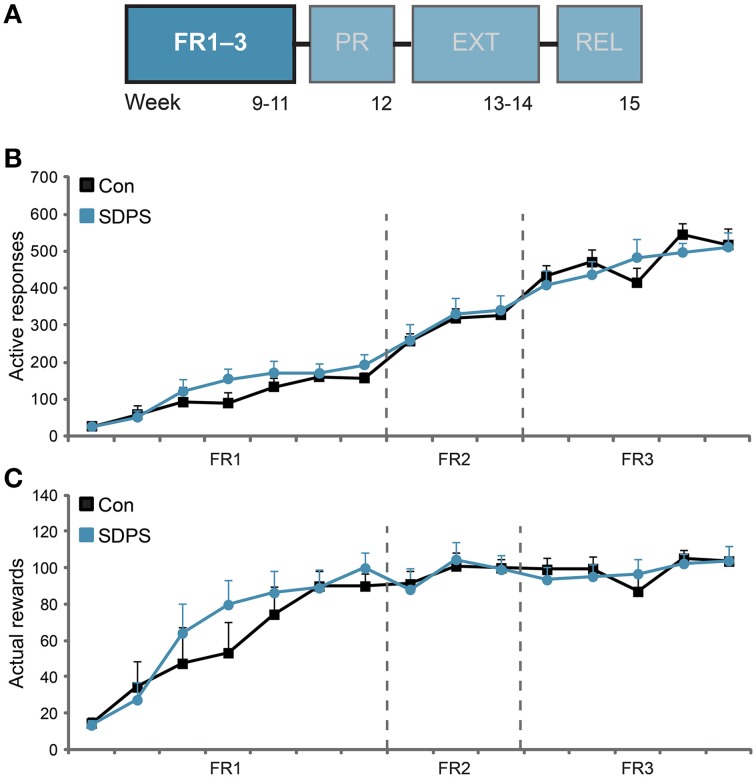
**SDPS does not affect acquisition of sucrose SA nor sucrose consumption**. **(A)** Animals participated at the operant sucrose self-administration (SA) paradigm, including 3 weeks of FR1–3 training schedules, 1 week of PR, 2 weeks of extinction and finally the relapse tests; FR1–3 training **(B,C)** is highlighted. **(B)** All animals increased responding to the sucrose delivery-associated hole, as function of the FR schedules examined, indicating consolidation of the task. SDPS had no effect on acquisition of sucrose SA, since SDPS and controls performed identical under all three ratios assessed. **(C)** No difference in the number of rewards obtained during acquisition of sucrose SA between SDPS and control rats were observed. Notably, both groups stabilized their sucrose intake after the introduction of FR2 training schedule, with a group average of ~100 rewards/session, corresponding to ~5 g/kg of sucrose.

Following assessment of the depressive-like state, training toward the acquisition of cue-paired operant sucrose self-administration (SA) commenced (Figure 2A). Based on previous studies with alcohol (Riga et al., 2014), a slightly modified protocol for sucrose SA was used, omitting home-cage taste familiarization.

#### Fixed ratio (FR)

Rats were trained to nose-poke for a 0.20 mL 12% sucrose reward in 1-h sessions provided every day. Sucrose delivery (US) was accompanied by discrete audiovisual stimuli (CS, 4-s active hole illumination and tone presentation). Initially, a continuous reinforcement (fixed ratio 1, FR1) schedule was implemented, in which each reward delivery was followed by a 15-s time-out period, during which nose poking has no programmed consequences. Responding on the inactive hole was monitored, but had no consequences. When FR1 performance reached stable levels (Criteria: (1) >50 rewards per 1-h session; (2) no statistically significant differences between the last 2 FR sessions, as assessed using repeated measures ANOVA), animals were introduced to FR2 and subsequently FR3 training schedules. Consolidation of sucrose SA was estimated by peak performance at FR3 (491 ± 39 active responses/1-h session for controls and 496 ± 40 for SDPS). Animals were trained under FR schedules for a total of 3 weeks.

#### Progressive ratio (PR)

Animals were subjected to eight daily 2-h progressive ratio (PR) sessions, during which the effort (number of nose-pokes) to obtain a reward was progressively increased according to: response ratio = (5e^(0.2 * *reward number*)^) −5, rounded to the nearest integer (Richardson and Roberts, 1996). Each session automatically ended when no reward was delivered (no FR was reached) within an hour. All animals completed the 2-h training sessions, independently of group.

#### Extinction and reinstatement

The day after the last PR session, all animals were re-trained to FR1 to minimize between-group differences that could affect subsequent analysis of extinction performance. Animals were provided with 2 daily 1-h FR1 training sessions before the onset of extinction training. Extinction consisted of 1-h exposure to the training context in absence of sucrose and sucrose-associated cues. Following nine daily sessions, operant responding was successfully extinguished as reflected in rats reaching < 5% of group-average FR1 responding by the last extinction session. All animals participated in a 30-min cue-induced reinstatement session, at the start of which a single 0.20 mL sucrose reward was delivered. Nose poking during the session resulted in presentation of the discrete compound audiovisual cues (but no sucrose reward) on an FR1 schedule. Reinstatement of sucrose seeking was calculated based on animals' performance during the last extinction session.

#### Guanfacine administration

Guanfacine-HCl [*N*-amidino-2-(2,6-dichlorophenyl) acetamide hydrochloride] was tested during PR sessions five and eight, given 3 days apart, as well as on cue-induced reinstatement in two separate tests, given at a 4-day interval without additional extinction training. Saline (1 mL/kg) or guanfacine (0.5 mg/kg dissolved in saline) were systemically (i.p.) administered 1 h before the session/test in a cross-over design.

All self-administration procedures took place in MED Associates INC® (St. Albans, VT, USA) operant behavior chambers, surrounded by sound-attenuating cubicles. Data were collected using the MED-PC software package.

#### Statistical analyses

All statistics were performed using SPSS (version 15.0, IBM) and data are presented as mean ± SEM. All behavioral data collected from SAA and OPR tests were analyzed using One-Way analysis of variance (ANOVA) with defeat as the between-subjects factor. Similarly, data collected during sucrose SA, including PR, relapse and guanfacine administration, were analyzed using mixed ANOVAs with defeat as the between-subjects factor and session (or, if applicable, treatment) as the within-subjects factor. When *P*-values reached level of significance (*P* < 0.05), further analysis was performed using One-Way ANOVA, paired or unpaired (*post-hoc*) Student's *t*-test. Homogeneity and equality of variance were estimated and Hyunh-Feldt or Levene's test corrections were implemented in case of assumption violation. All interaction or exploration indexes (SAA, OPR) were calculated based on animals' performance during the 1st minute of the test phase of each task. For the aforementioned tasks, preference indices (interaction or exploration) were based on a fictive group showing no discrimination, while retaining the variation of the tested sample (Akkerman et al., 2012).

## Results

### SDPS induces deficits in the affective and cognitive domains

Eight weeks following the last defeat episode we assessed the development of the depressive-like state on both affective and cognitive domains (Figure 1A). To examine the effects of SDPS on social behavior, the social approach-avoidance (SAA) test was employed (Figure 1B). All animals showed willingness to interact with the social target: Con, *t*_(6)_ = 14.42, *P* < 0.001; SDPS, *t*_(7)_ = 4.23, *P* = 0.004 vs. *fictional*). However, defeated rats exhibited a significant decrease in interaction time as compared with controls: *t*_(12.39)_ = 2.61, *P* = 0.022. Similarly, cognitive performance was impaired in the SDPS group, as assessed using the object place recognition (OPR) test (Figure 1C). In particular, SDPS animals displayed deficits in retention of an object's spatial location when compared with controls: *t*_(13)_ = 2.54, *P* = 0.025. Taken together, these results pointed to the establishment of a depressive-like state that persists over time and that mimics core phenotypic manifestations of the human disorder (Austin et al., 2001; Millan et al., 2012).

### SDPS increases motivation for a natural reward

#### Acquisition of operant sucrose self-administration

Animals were subjected to seven FR1, three FR2 and five FR3 training sessions (Figure 2A). Overall, SDPS did not affect responding for a sucrose reward under any of the schedules investigated (Figure 2B). All animals learned to discriminate between the active and the inactive hole from the first training session onwards [Con, *t*_(6)_ = 2.80, *P* = 0.031 at FR1 session 4; SDPS, *t*_(7)_ = 3.27, *P* = 0.014 at FR1 session 3]. In FR1 schedule, repeated measures ANOVA revealed a significant effect of training on active responding [*F*_(3.67, 47.75)_ = 17.36, *P* < 0.001], as all animals increased performance over time. No effect of group × training was observed, indicating similar FR1 acquisition in both controls and SDPS: *F*_(3.67, 47.75)_ = 0.88, *P* = 0.474. Indeed, no between-group differences in responding were found: *F*_(1, 13)_ = 0.75, *P* = 0.402. Similarly during FR2, an overall increase in the number of active responses was observed [*F*_(2, 26)_ = 8.97, *P* = 0.001], which was independent of group: group × training, *F*_(2, 26)_ = 0.04, *P* = 0.958. As with FR1, no between-group differences in acquisition were observed in FR2: *F*_(1, 13)_ = 0.04, *P* = 0.850. FR3 further increased animals responding on the active hole over time [*F*_(4, 52)_ = 7.03, *P* < 0.001], independently of group: group × training, *F*_(4, 52)_ = 1.88, *P* = 0.128. As with preceding training schedules, no significant differences between controls and defeated animals were found: *F*_(1, 13)_ = 0.03, *P* = 0.858.

With respect to the number of rewards gained per session, controls and SDPS animals showed similar performance in each of the FR training schedules employed (Figure 2C): *FR1*: training, *F*_(3.38, 43.93)_ = 29.67, *P* < 0.001; group × training, *F*_(3.38, 43.93)_ = 1.11, *P* = 0.358; group *F*_(1, 13)_ = 0.35, *P* = 0.566; *FR2*: training, *F*_(1.65, 21.49)_ = 2.73, *P* = 0.096; group × training, *F*_(1.65, 21.49)_ = 0.17, *P* = 0.807; group *F*_(1, 13)_ = 0.00, *P* = 0.993; *FR3*: training, *F*_(4, 52)_ = 3.37, *P* = 0.016; group × training, *F*_(4, 52)_ = 1.20, *P* = 0.321; group *F*_(1, 13)_ = 0.04, *P* = 0.949. Accordingly, at the end of the FR training period, animals consumed considerable, but equivalent, amounts of the sweetened water solution: Con, 19.7 ± 1.4 mL (~4.7 g/kg); SDPS, 20.2 ± 1.4 mL (~4.9 g/kg) per hour.

SDPS and control rats performed identical for inactive responses, as both groups reduced responding over time in a similar rate (Supplementary Figure 1A). Time-out responses increased over time during all training schedules, but no between-group differences were observed (Supplementary Figure 1B). Taken together, the FR data showed that SDPS did not alter acquisition of sucrose self-administration nor sucrose intake as compared with controls.

#### Progressive ratio (PR) and guanfacine treatment

**Figure 3:**
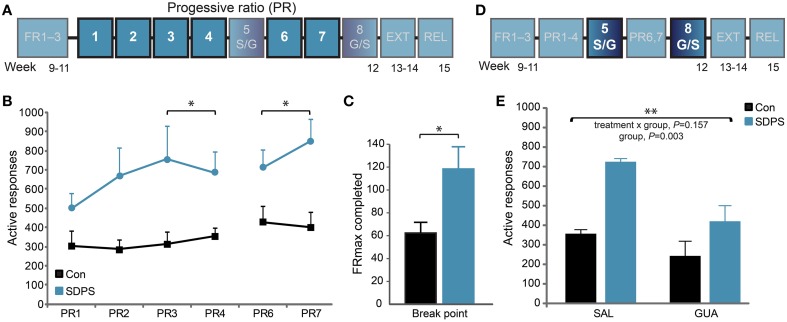
**SDPS triggers motivational overdrive toward a sucrose reward; guanfacine attenuates enhanced motivation. (A)** The motivation to acquire a sucrose reward was examined in 6 treatment-free PR training sessions (highlighted; **B,C**). **(B)** SDPS increased responding during both the pre- (PR3–4) and post- (PR6–7) treatment sessions, and no carry-over effects of guanfacine treatment were detected (see **E**). **(C)** Break points, depicted as the maximum FR completed when averaging over the four treatment-free sessions, confirmed the SDPS-triggered exaggeration of motivational drive in defeated animals. **(D)** The effect of guanfacine on PR responding was assessed at PR sessions five and eight using a cross-over treatment administration design (highlighted, **E**). **(E)** Guanfacine administration reduced overall PR responding. Independently of treatment regime, the SDPS group showed significantly increased number of responses as compared with controls. ^*^P < 0.050; ^**^P < 0.010; **(B)**: repeated ANOVA (group effect); **(C)**: One-Way ANOVA (group effect); **(E)**: repeated ANOVA (treatment effect).

The PR training schedule was introduced to examine SDPS-triggered differences in motivation towards sucrose acquisition (Figure 3A). Repeated measures ANOVA over the last 2 PR sessions before the first guanfacine challenge (PR3–4) showed no training effect [*F*_(1, 13)_ = 0.05, *P* = 0.831], nor a training × group interaction [*F*_(1, 13)_ = 0.65, *P* = 0.433], indicating stabilization of PR performance. Notably, a significant group effect was observed [*F*_(1, 13)_ = 6.68, *P* = 0.023], as SDPS animals showed enhanced responding for sucrose when compared with controls (Figure 3B). Similar to active responses, break points, calculated based on the highest FR completed (average of treatment-free sessions PR3–4 and PR6–7), were significantly increased following SDPS [*F*_(1, 14)_ = 6.15, *P* = 0.028] pointing toward heightened motivation to acquire sucrose (Figure 3C).

As guanfacine, an α_2_-adrenergic agonist, has been shown to reverse SDPS effects on PR-responding for alcohol (Riga et al., 2014), we questioned whether it would also be beneficial against the increased motivation for sucrose intake. Guanfacine was administered in two separate PR sessions (PR sessions 5 and 8), in a cross-over design (Figure 3D). Repeated measures ANOVA revealed that pretreatment with guanfacine (GUA), 1 h before the session, reduced active responding for sucrose independently of defeat, compared with saline (SAL) treated animals (Figure 3E, Supplementary Figure 2): treatment, *F*_(1, 13)_ = 11.44, *P* = 0.005; group × treatment, *F*_(1, 13)_ = 2.26, *P* = 0.157. Importantly, a significant group effect was observed [*F*_(1, 13)_ = 12.96, *P* = 0.003], as SDPS rats showed enhanced performance at both PR sessions. Guanfacine effects did not carry-over in-between treatment days, as a similar group effect observed prior to guanfacine treatment on PR responding was present when analyzing sessions after guanfacine treatment [PR6–7: *F*_(1, 13)_ = 7.57, *P* = 0.017].

#### Extinction of operant sucrose self-administration

**Figure 4:**
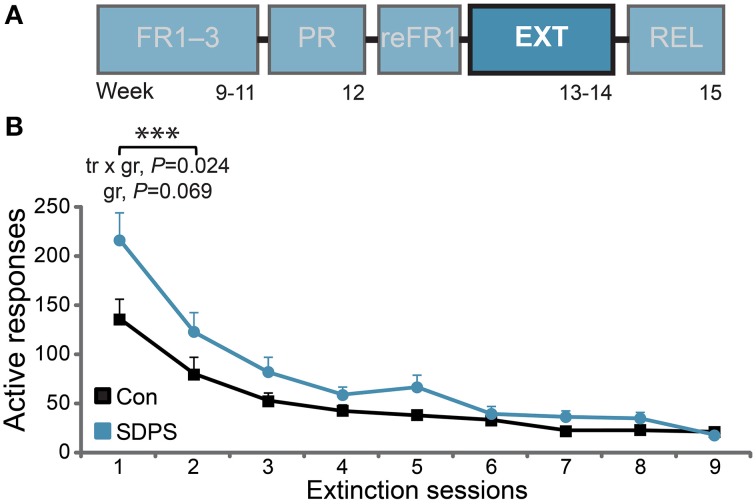
**SDPS hinders extinction of sucrose SA**. **(A)** Following retraining to FR1 (see Supplementary Figure 3), all animals were subjected to extinction of the sucrose-delivery context, in nine daily sessions (highlighted). **(B)** Responding to the active hole stabilized in controls following the first two sessions, indicating extinction of the context conveying sucrose availability. A significant training × group (tr × gr) interaction and a trend for group (gr) effect indicate initial resistance to extinction displayed by the SDSP animals. ^***^P < 0.001; repeated ANOVA (training effect).

Following PR, all animals were re-trained under FR1 schedule (reFR1), in order to restore similar between-group performance before proceeding with extinction training (Figure 4A). Already from the first reFR1 session, SDPS and control rats showed identical responding, at a similar level as during FR acquisition (Supplementary Figure 3). This was repeated at the second reFR1 session provided: repeated measures ANOVA; *reFR1* active responses: training, *F*_(1, 13)_ = 0.16, *P* = 0.693; group × training, *F*_(1, 13)_ = 1.92, *P* = 0.189; group *F*_(1, 13)_ = 0.06, *P* = 0.803; *reFR1* rewards: training, *F*_(1, 13)_ = 0.00, *P* = 0.977; group × training, *F*_(1, 13)_ = 0.19, *P* = 0.666; group *F*_(1, 13)_ = 0.51, *P* = 0.489]. Together reFR1 data confirmed that SDPS effects on PR-responding were not carried over to subsequent reinforcement schedules, and that PR training did not influence consummatory approach toward a sucrose solution.

Animals were then subjected to extinction of the context of reward delivery in nine daily sessions (Figure 4A). Analysis of the first two extinction sessions, during which sucrose unavailability was consolidated in controls, revealed a significant training effect [*F*_(1, 13)_ = 106.60, *P* < 0.001], as both groups reduced responding to the active hole over time (Figure 4B). Importantly, a significant training × group interaction was observed [F_(1, 13)_ = 6.54, *P* = 0.024], as the initial rate of responding in SDPS rats was higher in comparison with controls. This was partially confirmed by a trend for group effect: *F*_(1, 13)_ = 3.93, *P* = 0.069. By extinction session nine, both groups responded at < 5% of their initial (reFR1) performance: Con, 4.3 ± 0.8 and SDPS, 3.4 ± 0.8 responses per hour. These data indicate that, although SDPS rats showed an initial resistance to extinction learning, they could be extinguished to control levels following multiple extinction sessions.

#### Reinstatement and the effect of guanfacine treatment

**Figure 5:**
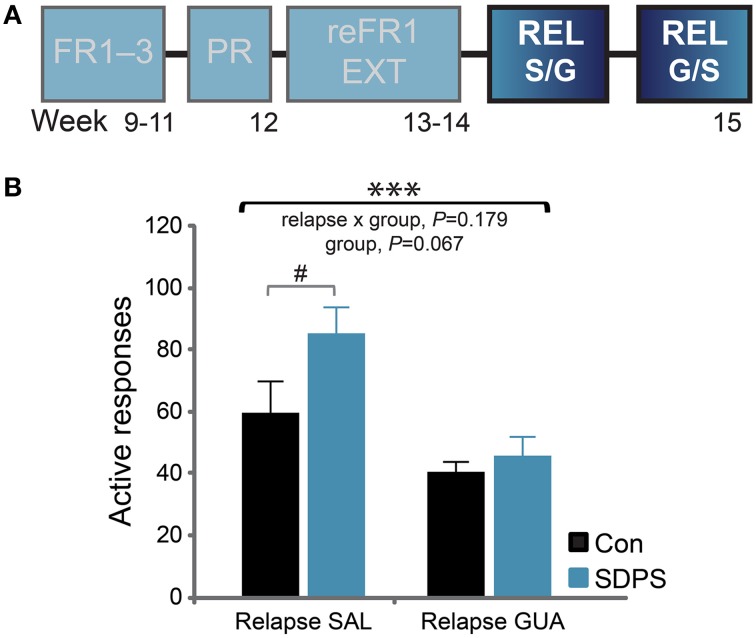
**SDPS mildly affects reinstatement of sucrose SA; guanfacine limits relapse. (A)** Following extinction, the ability of the sucrose-coupled cues to reinstate active responding was assessed in two relapse tests, given at 4 days interval, using a cross-over treatment administration design (highlighted). **(B)** SDPS mildly affected cue-induced relapse (left); after guanfacine treatment, relapse to sucrose seeking was limited in both groups (right), as shown by a significant treatment effect. A trend for group differences was observed, as SDSP animals responded at a relatively higher rate when compared with controls during the SAL session; this difference was no longer detected following treatment (GUA). ^#^*P* = 0.069; *post-hoc* (group effect); ^***^P < 0.001; repeated ANOVA (treatment effect).

Cue-induced reinstatement of sucrose seeking was assessed in two separate 30-min relapse tests (REL). Using a cross-over design, rats were systemically administered either guanfacine (GUA) or saline (SAL) 1 h before the test (Figure 5A). During the saline session, the presentation of cues that were previously associated with the delivery of sucrose was sufficient to reinstate responding in both groups, as compared with their own extinction performance: repeated measures ANOVA for relapse SAL: *F*_(1, 13)_ = 83.13, *P* < 0.001. A significant relapse x group interaction, [*F*_(1, 13)_ = 4.87, *P* = 0.046] and a trend for a group effect [*F*_(1, 13)_ = 2.92, *P* = 0.111] indicated that SDPS moderately increased reinstatement of sucrose seeking. *Post-hoc* analysis confirmed that both groups showed a significant effect of reinstatement as compared with their own extinction performance [Con, *t*_(6)_ = −4.52, *P* = 0.004; SDPS, *t*_(7)_ = −9.25, *P* < 0.001]. Yet, in the reinstatement session, SDPS animals showed a trend for higher seeking behavior than controls [Relapse SAL, *F*_(1, 14)_ = 3.93, *P* = 0.069; Supplementary Figure 4].

During the guanfacine session, both groups showed a significant increase in responding vs. their own extinction performance: repeated measures ANOVA for relapse GUA: *F*_(1, 13)_ = 32.20, *P* < 0.001. No relapse × group interaction [*F*_(1, 13)_ = 0.00, *P* = 0.955], nor group effects [*F*_(1, 13)_ = 1.15, *P* = 0.303] were observed confirming that guanfacine did not completely prevent reinstatement of sucrose seeking in either group. Importantly, a significant treatment effect was shown between the 2 relapse days (Figure 5B): repeated measures ANOVA for treatment: *F*_(1, 13)_ = 16.85, *P* = 0.001. This was accompanied by a non-significant treatment × group interaction [*F*_(1, 13)_ = 2.01, *P* = 0.179] and a trend for a significant group effect: *F*_(1, 13)_ = 3.99, *P* = 0.067]. Taken together, our data indicate that guanfacine pretreatment significantly reduced reinstatement of sucrose seeking, and that this reduction in responding seemed more prominent in the SDSP group.

## Discussion

The depressive state is characterized by multifaceted behavioral manifestations that span from negative mood and suicidality to indecisiveness, cognitive confusion, and blunted emotional reactivity (Leistedt and Linkowski, 2013). Animal models aiming to mimic depressive disorders are frequently impeded by such phenotypic complexity. In this study we showed that SDPS, an animal paradigm that mimics a sustained depressive state, is able to induce deficits in processing of affective information and reward evaluation, similar to that observed in patients. Furthermore, we showed that guanfacine, an FDA-approved agent that reverses SDPS-induced excessive alcohol seeking (Riga et al., 2014), rescues the anhedonia-like phenotype.

### SDPS-induced impairments in the affective and cognitive domains

In line with literature (Blanchard et al., 2001; Nestler and Hyman, 2010) a significant and sustained decline in approach behavior develops following SDPS (Riga et al., 2014). Social withdrawal is considered to be one of the most robust behavioral readouts in the field. It is employed to assess the magnitude of the depressive state, e.g., susceptibility vs. resilience (Krishnan et al., 2007) and to identify depression-mediating brain circuitries, as well as novel therapeutic approaches (Bruchas et al., 2011; Fanous et al., 2011). In our study, the duration of avoidance of the social target, long after the last exposure to a resident, is indicative of an established depressive state that persists well-beyond the actual stress incidence, mimicking clinical observations of MDD.

Apart from impairments in the affective domain, cognitive dysfunction, including poor working memory, mnemonic deficits, and impaired concentration [DSM 5th edition, (American Psychiatric Association, 2013)] is a hallmark of depression. Almost half of the depressed population exhibits deficits in at least one cognitive domain, with working memory and attention-related deficits most consistently reported (McIntyre et al., 2013). These deficits complement the depressive (endo)phenotype, exaggerating difficulties in decision-making and other aspects of executive control (Hasler et al., 2004; Murrough et al., 2011; Millan et al., 2012). In this study, we confirmed previous observations that SDPS triggered lasting impairments in short-term spatial memory (Riga et al., 2014) analogous to depression-induced cognitive impairments that characterize the enduring state of depression (Femenia et al., 2012).

### SDPS promotes the development of an anhedonia-like phenotype

Blunted responses to affective stimuli have been demonstrated consistently in depressed patients (Eshel and Roiser, 2010; Disner et al., 2011), including dysfunctional reward-related learning (Vrieze et al., 2013), reduced primary hedonic responses (Pizzagalli et al., 2008; McCabe et al., 2009), untenable connectivity between reward structures (Heller et al., 2009), decreases in reward anticipation and poorly integrated positive feedback (Smoski et al., 2009). These findings implicate impairments of affective cognition and reward processing in the development of depressive disorders. Similarly, direct links between behavioral anhedonia and the activity of reward-mediating systems (Keedwell et al., 2005; Schlaepfer et al., 2008) exemplify the strong association between dysfunctional reward processing and the magnitude of the depressive state.

Preclinically, the concept of anhedonia was, for many years, confined to findings of reduced appetitive interest or reduced preference for naturally rewarding items (e.g., sucrose). This has been consistently portrayed as representative of depression-induced deficits in behavioral reinforcement, an animal-equivalent of anhedonia. Clinically, anhedonia is nowadays not valued as a steady-state depressive symptom linked to an absolute “hedonic capacity,” but rather encompassing a much broader spectrum of consummatory and motivational deficits, involving reward-related decision-making and goal-directed behavior (Treadway and Zald, 2011; Der-Avakian et al., 2014). As reviewed by Der-Avakian and Markou (2012), anhedonia extends beyond the loss of feeling pleasure and conveys failure in (i) reward anticipation or prediction; (ii) estimation of the value-to-cost ratio for a given reward; (iii) assessment of reward accessibility as function of the effort required; (iv) integration of this information to justify (or oppose to) reward acquisition; and finally (v) motivation to act toward reward acquisition. Thus, anhedonia is a progressively developing phenotype that cannot be solely based on measurements of preference for a given reward and should include the contributions of motivational approach and reward-related learning and decision-making (Ho and Sommers, 2013).

In accordance with this updated conceptualization of anhedonia, the main aspect described in the present study concerns the ability of SDPS animals to evaluate a given reward and thus to modulate their behavior as function of reward-signaling information in the long-term. This is reflected in altered progressive ratio responding, extinction and reinstatement of sucrose seeking. SDPS increased break points under PR training schedules, a putative measurement of motivational drive (Hodos, 1961) and promoted inelastic demand (Diergaarde et al., 2012), as depicted in a clear shift in demand curve (Supplementary Figure 5). Additionally, SDPS affected extinction learning as SDPS rats showed a reduced capacity to incorporate new information signaling sucrose unavailability. Finally, SDPS rats showed a relative increase in reinstatement of sucrose seeking as compared with controls, reflecting maladaptive processing of reward-related information, and excessive reactivity to reward-paired cues. These deficits do not likely result from a general decline in learning capacity following SDPS, as SDPS rats and controls showed similar discriminative ability at the start of SA training (active vs. inactive hole) and similar SA acquisition rates at FR1–3. Moreover, SDPS rats did not show slower extinction of the alcohol-associated context (Riga et al., 2014), indicative of specificity for the type of reward.

Previously, we observed a similar, even exaggerated, phenotype for SDPS-induced motivation and relapse of operant alcohol intake (Riga et al., 2014). In accordance, intermittent social defeat stress increases PR operant responding for cocaine and cocaine binging (Covington and Miczek, 2005; Covington et al., 2005). In contrast, chronic defeat stress (~5 weeks of daily defeat sessions) induces a remarkable decrease in both PR responding and cumulative cocaine intake for up to 5 weeks following the last defeat session (Miczek et al., 2011). It is noteworthy that different types of social stress persistently alter the incentive value of a given reward, although the direction of changes in motivational drive depends on the stress-status of the animal. Indeed, and in contrast to the lasting effect of SDPS, acute defeat results in decreased motivational drive, as expressed in reduced PR saccharin-reinforced responding (Miczek et al., 2011) and decreased operant alcohol self-administration (Funk et al., 2005). We previously reported that SDPS-induced social withdrawal can predict higher motivation to seek and consume alcohol (Riga et al., 2014). In the current study, such correlations between approach-avoidance behavior and motivational deficits were not observed, suggesting a differential effect of SDPS on motivation for natural vs. non-natural rewards (Supplementary Figure 6).

### Guanfacine attenuates the SDPS-induced anhedonia-like phenotype

Guanfacine is a selective α_2_-adrenergic agonist recently FDA-approved for the treatment of attention deficit hyperactivity disorder (ADHD) (Muir and Perry, 2010). Owing to its cognitive enhancing properties (Sofuoglu et al., 2013), guanfacine has been used in both clinical and pre-clinical settings to limit stress- and cue-induced drug-seeking and craving (Lee et al., 2004; Smith and Aston-Jones, 2011; Fox et al., 2012, 2015; Fredriksson et al., 2015; McKee et al., 2015). In addition, we recently showed that a comparable dose of guanfacine prevents SDPS-induced motivational overdrive and susceptibility to cue-induced relapse in an alcohol self-administration paradigm (Riga et al., 2014). In naïve rats, the effects of guanfacine against stress-facilitated reinstatement of food pellets showed large inter-individual variability (Le et al., 2011), making it of great interest to examine whether it can selectively reverse the anhedonia-like phenotype as seen after SDPS. In the present study, guanfacine was used against SDPS-triggered increases in PR-responding and in cue-paired reinstatement of sucrose seeking. Guanfacine pretreatment was sufficient to reduce overall PR responding, but these effects were independent of the depressive phenotype, as they were observed in both control and SDPS rats. Although it did not completely abolish the increase in break points, SDPS PR responding decreased considerably after guanfacine, indicating a beneficial effect against depression-related pathological manifestations, such as maladaptive motivational drive. Furthermore, whereas guanfacine pretreatment reduced cue-induced reinstatement of sucrose-seeking in both groups, it limited the magnitude of SDPS relapse to control levels. Together, these results pinpoint to SDPS-specific effects of guanfacine, and identify a novel property of the drug, as being beneficial against the depression-induced anhedonia-like phenotype.

### Methodological considerations

Given that anhedonia is a key feature of clinical depression, animal models aiming to explore this complex disorder should employ some kind of assessment of reward deficits and (diminished) reinforcement (Anisman and Matheson, 2005). To this end, several different behavioral readouts have been established over the years to emulate depression-induced anhedonia. Amongst them, sucrose-based (preference or anticipation) paradigms have been most extensively used, although yielding contradictory results (Der-Avakian et al., 2014).

In particular, varying findings, i.e., increases, decreases or unaltered behavior, of the effects of stress on sucrose consumption or preference have been reported depending on the models applied, including differences in species, strain (Nielsen et al., 2000; Pothion et al., 2004; Henningsen et al., 2009; Razzoli et al., 2011) and gender (Bai et al., 2014), type of stressors (non-social or social), timing and duration of stress (Meerlo et al., 1996; Rygula et al., 2005; Krishnan et al., 2007; Miczek et al., 2011; Muto et al., 2014), food availability (Forbes et al., 1996; Barr and Phillips, 1998), and sucrose concentrations employed (Bondar et al., 2009; Hollis et al., 2011). In the majority of studies, the most robust changes in sucrose intake are observed during or acutely following chronic stressors, whereas these changes quickly recover following termination of stress exposure. Few studies examined the after-effects of social stress on anhedonia, reporting reduced sucrose consumption up to 3 weeks following defeat (Becker et al., 2008; Carnevali et al., 2012) or unaltered intake at 9–11 weeks following the last defeat encounter (Von Frijtag et al., 2000).

Together, a large body of evidence indicates that differences in sucrose preference can be used to assess acute effects of social defeat or short-lasting effects after chronic defeat stress exposure in rodents. In addition, these findings illustrate that assessment of sucrose intake is not suitable to estimate the anhedonia-like phenotype during the maintenance phase of a depressive state (Von Frijtag et al., 2000; Kamal et al., 2010). This is in accordance with our current data, since SDPS rats, although in a stable, sustained depressive-like state reflected by maladaptive motivational drive, did not display any alteration in sucrose intake during acquisition or re-training at FR1, as compared with controls. To our knowledge, this is the first study reporting long-lasting anhedonia-like behaviors as assessed by instrumental responding for a natural reward. Importantly, in our study, by incorporating motivation, extinction and reinstatement into the assessed behavioral repertoire, we identified novel readouts affected by the depressive state and we advanced our understanding on what is, and how we can measure, anhedonia at the preclinical level.

Highly palatable food, such as items containing sugar or fat, induces stable preference and elevated consumption in rodents (Hone-Blanchet and Fecteau, 2014). In our study, the intake during FR training schedules was identical between control and defeated animals, implying that both groups estimated the caloric value of sucrose equally. Thus, the increased responding observed in PR training in SDPS animals cannot be attributed to differences in “liking” or taste. Similarly, differences in total fluid intake cannot account for the SDPS effects on sucrose seeking, as SDPS animals display similar water intake as controls, from the week following the defeat week onwards (Supplementary Figure 7). It is noteworthy that, contradictory findings have been reported when employing a different animal model of depression, i.e., the chronic mild stress (CMS) paradigm, depending on the concentration of sucrose. In particular, CMS leads to enhanced sucrose solution intake in higher concentrations (30–40%), whereas it suppresses intake of lower sucrose concentrations (1–2%) (Willner, 1997). We consider that at the intermediate 12% used here, sucrose solution remains highly palatable and at an optimal concentration for intake (Barr and Phillips, 1998; Pothion et al., 2004).

Unspecific motor effects of guanfacine, such as sedation, could not account for the reduction in responding displayed by controls and SPDS rats in either PR or relapse test, as previously shown (Riga et al., 2014). Indeed, upon guanfacine treatment rates of responding for sucrose remained high in both groups at PR training (Figure 3) and at relapse (Figure 5). Similarly, guanfacine did not affect PR inactive responding, further excluding non-specific effects on motor function (Supplementary Figure 8). It should be noted that guanfacine-induced suppression of responding was more robust in the defeated animals at both PR training and relapse tests, whereas in the latter, guanfacine was sufficient to normalize the enhanced SDPS responding back to control levels.

In the present study, the established depressive state led to maladaptive motivational drive and vulnerability to sucrose-associated context and cues. Although SDPS animals and controls showed similar consummatory behavior toward a presumably enticing sucrose solution, during PR training SDPS rats failed to appreciate the relative effort-to-outcome relationship and displayed inelastic demand when reward was delivered at higher costs. During extinction training, depressed animals exhibited delayed uncoupling of the context of sucrose delivery from the reward itself, indicative of dysfunctional processing of novel reward-associated information. Finally, SDPS-induced proneness to reinstatement of sucrose seeking confirmed an excessive increase in the incentive salience of reward-signifying cues. Taken together, these effects are in accordance with the vast majority of literature on the human depression-associated anhedonia, describing maladaptive integration of learned and retrieved reward-coding information and, consequently, misguided behavioral adaptations in response to reward-related stimuli (Pizzagalli et al., 2008; Eshel and Roiser, 2010; Vrieze et al., 2013). Similarly our results reflect a large share of clinical observations linking anhedonia and depression with motivational deficits that correspond better to impaired reward-associated anticipation, appraisal, and decision-making rather than diminished consummatory and/or experiential hedonic responses (Chentsova-Dutton and Hanley, 2010; Padrao et al., 2013).

## Funding and disclosure

AS and SS received partial funding by the Center for Medical Systems Biology (CMSB). DR is partly funded by an NCA proof-of-concept fund (SS). None of the other authors have a present or anticipated employment, conflicts of interest, financial or otherwise, related to the subject of the reported findings or that is affected by its publication.

### Conflict of interest statement

The authors declare that the research was conducted in the absence of any commercial or financial relationships that could be construed as a potential conflict of interest.
