# Supplementary material for: Social defeat-induced anhedonia: effects on operant sucrose-seeking behavior
Source: Front Behav Neurosci. 2015 Aug 7;9:195. doi: 10.3389/fnbeh.2015.00195 (PMC4528167; doi:10.3389/fnbeh.2015.00195)
Supplement: Supplementary file 1 [file DataSheet1.DOCX]

**Supplemental Figures**


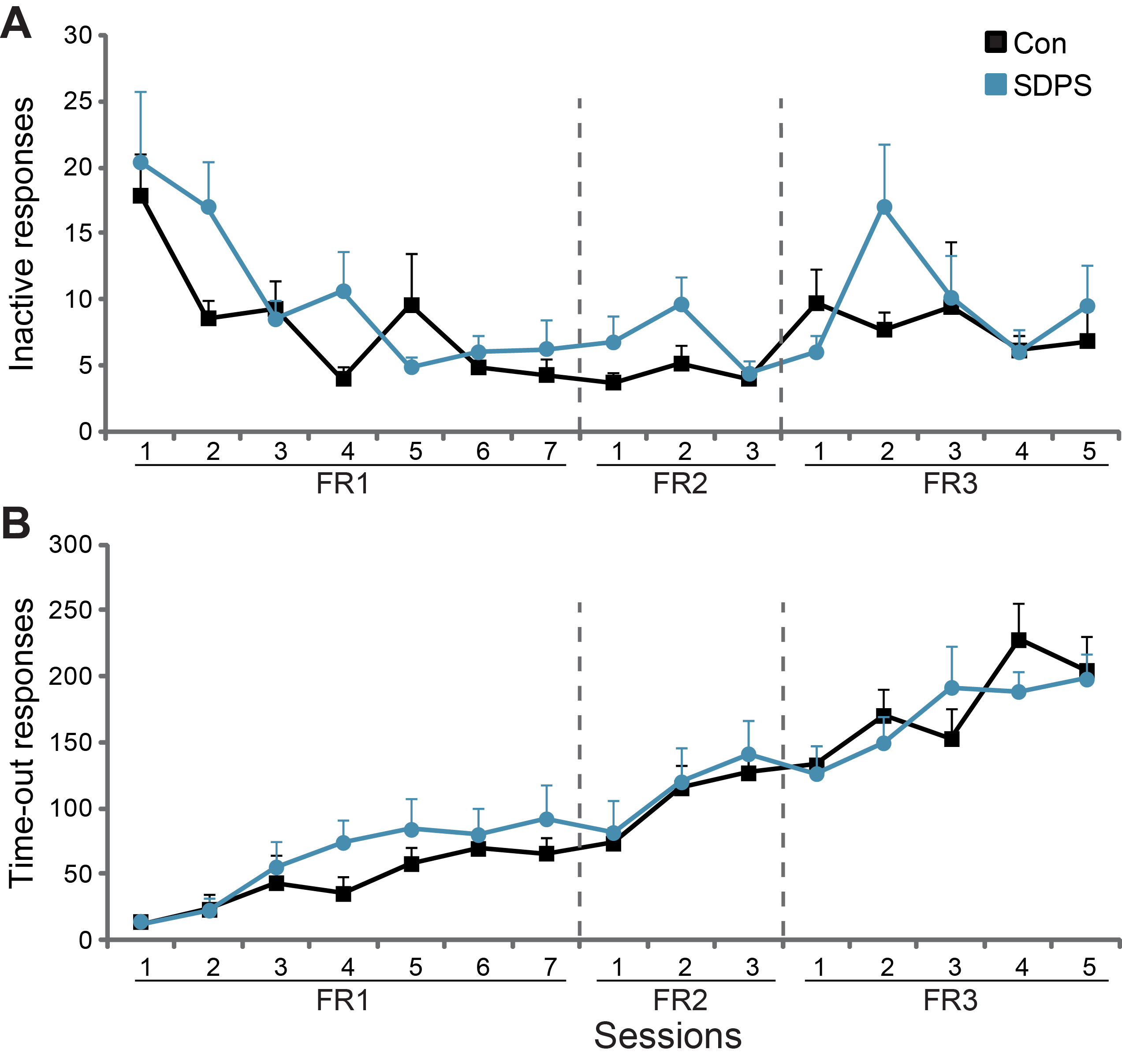


**Supp. Fig. 1.** **SDPS does not affect inactive or time-out responding during FR1–3 training.** A) During acquisition of sucrose self-administration no group differences between control and SPDS rats were detected at any of the FR training schedules employed for the number of inactive responses. These decreased during FR1 and stayed low for the remaining training period, indicating that all rats learned to preferentially respond to the active, sucrose-delivering hole. B) Whereas the number of time-out responses progressively increased as function of the various FR training schedules introduced, no differences between controls and SDPS rats were observed.


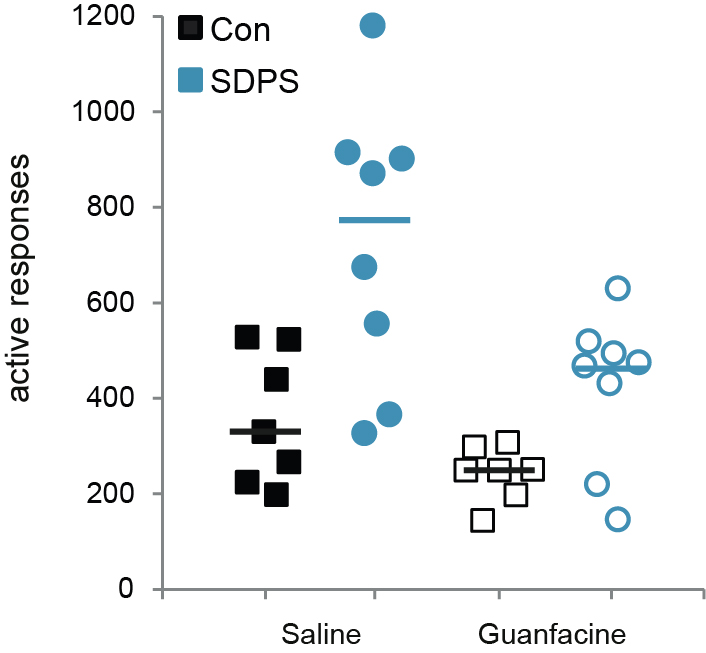


**Supp. Fig. 2.** **Individual performance during the two PR-treatment sessions.** Progressive ratio responding increased significantly by SDPS in the saline session, similar to what seen during the non-treatment days. Guanfacine administration reduced PR responding in both groups, but this effect was considerably larger in the SDPS group. Open squares/circles depict individual performance at the guanfacine PR session. Horizontal lines depict group medians during the two sessions.


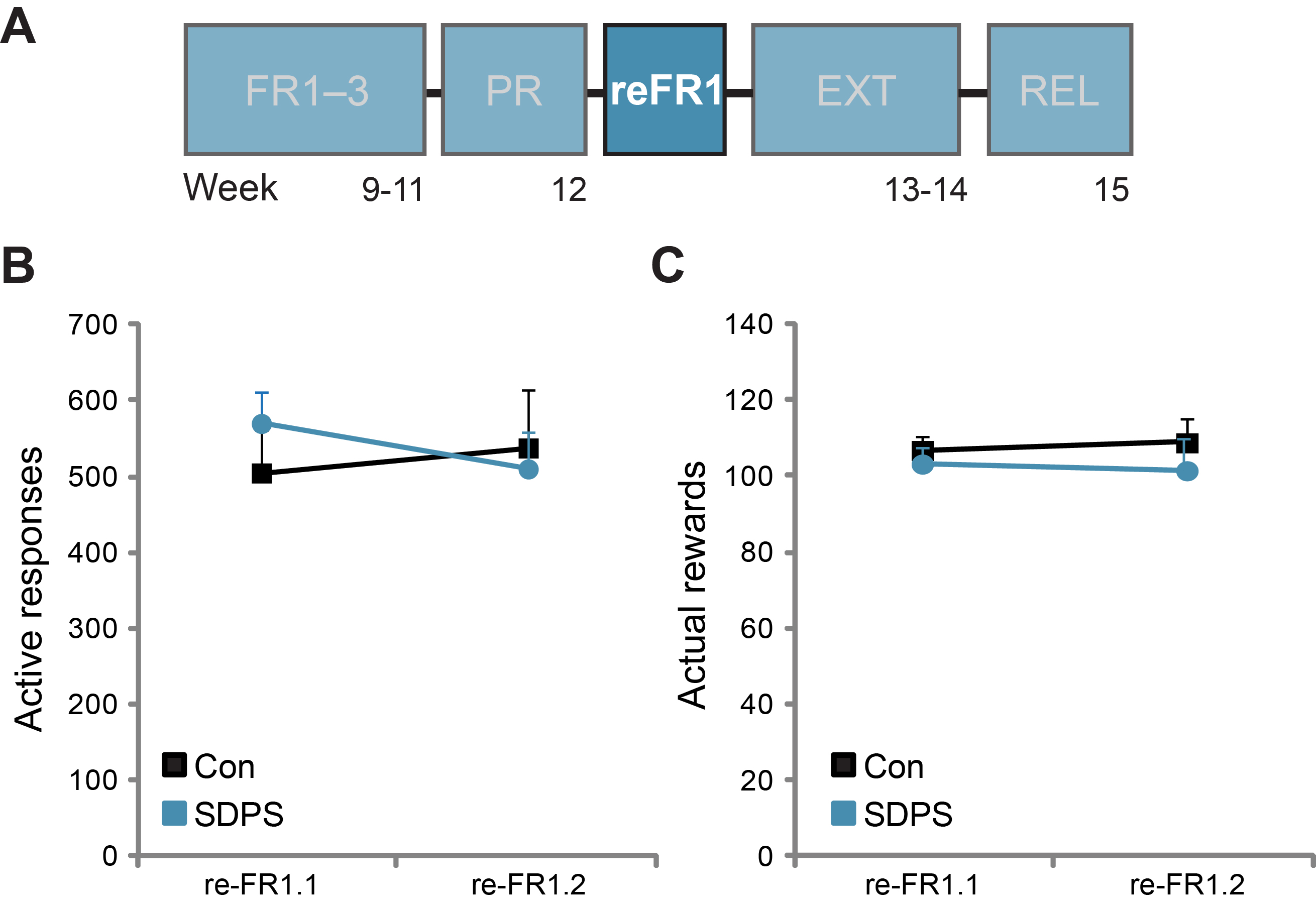


**Supp. Fig. 3.** **SDPS effects on PR responding do not carry over when retraining at FR1.** A) Following training under PR schedule, all animals were retrained to FR1 (reFR1), in order to prevent pre-existing between-group differences impeding extinction performance (highlighted). To this end, two FR1 sessions were introduced. B,C) Similar to what observed during acquisition, SDPS did not affect the number of active responses (B) or rewards obtained (C) during the two FR1 sessions. Notably, sucrose consumption remained at the same stable levels as during initial acquisition training.


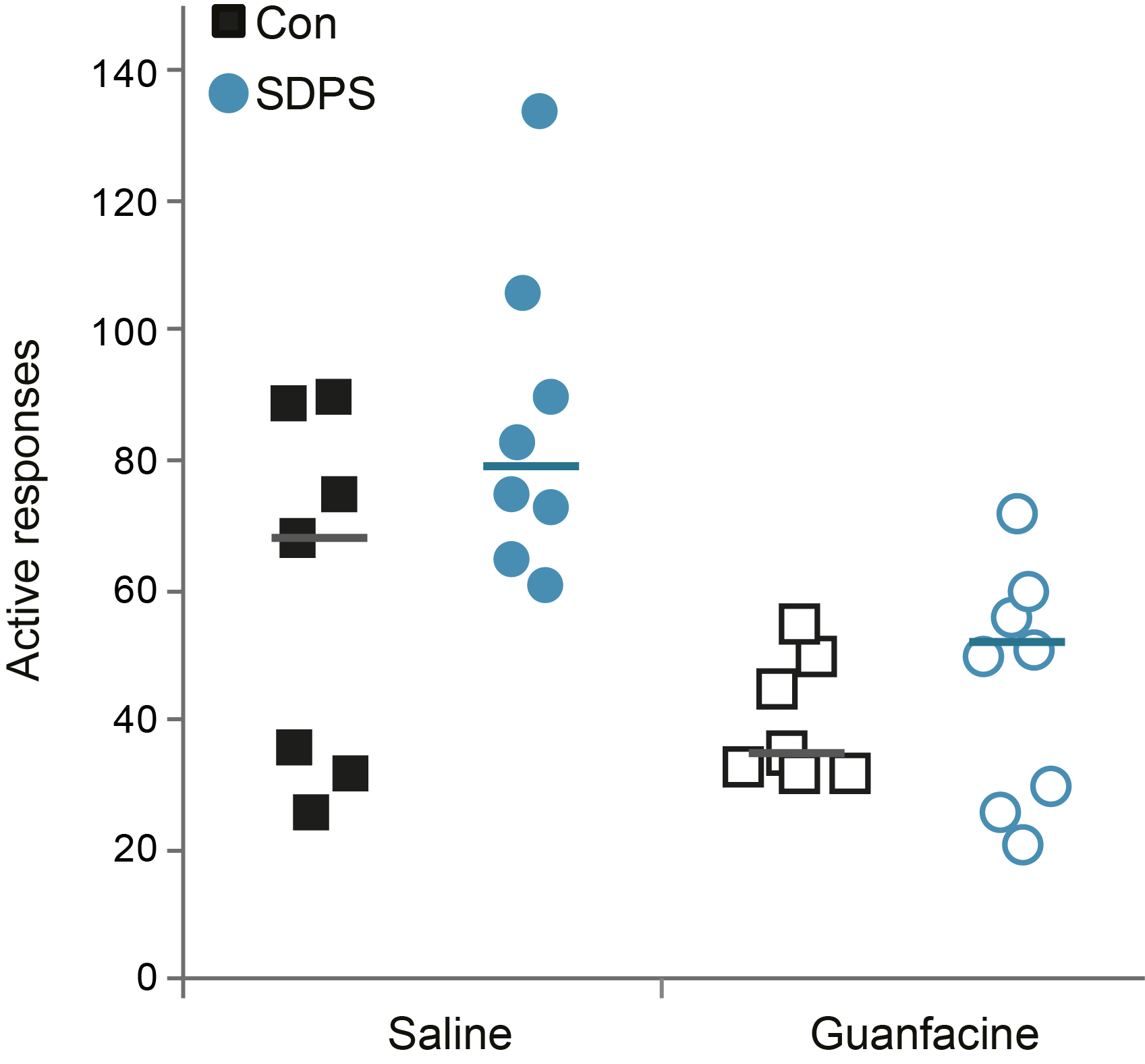


**Supp. Fig. 4.** **Individual performance during the two relapse tests.** Following presentation of the sucrose-coupled cues, both SDPS and control groups reinstated sucrose-seeking behavior, independently of treatment (saline *vs.* guanfacine). SDPS induced a non-significant increase in responding at the saline-test as compared to controls, however, the low n-number as well as the large within-group variation, occludes these results from being conclusive. Open squares/circles depict individual performance at the guanfacine relapse test. Horizontal lines depict group medians during the two tests.


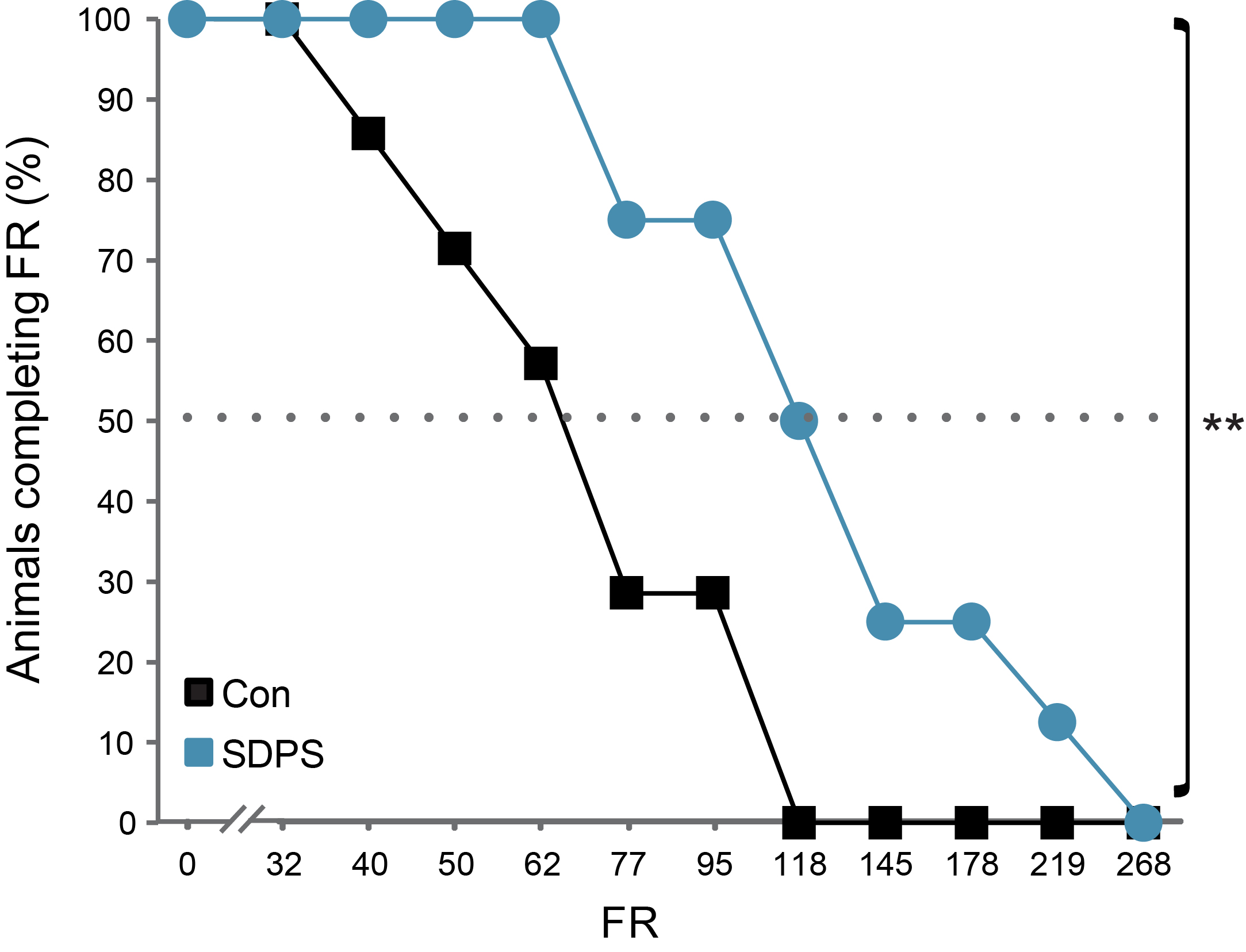


**Supp. Fig. 5.** **SDPS induces behavioral inflexibility towards a sucrose reward.** PR data are depicted as % of animals reaching correspondent FR schedule (average of 6 treatment-free sessions). As seen by a significant rightward shift in the demand curve, SDPS induced inelastic demand, so as a larger percentage of SDPS animals continued to respond for a sucrose reward even when its delivery required the acquisition of high FR schedules. ** *P*=0.015. Dotted line represents 50% cut-off, when the majority of animals stop responding for a given reward due to the high reward-to-effort ratio. Statistical differences were calculated based on Kaplan-Meier survival estimator.


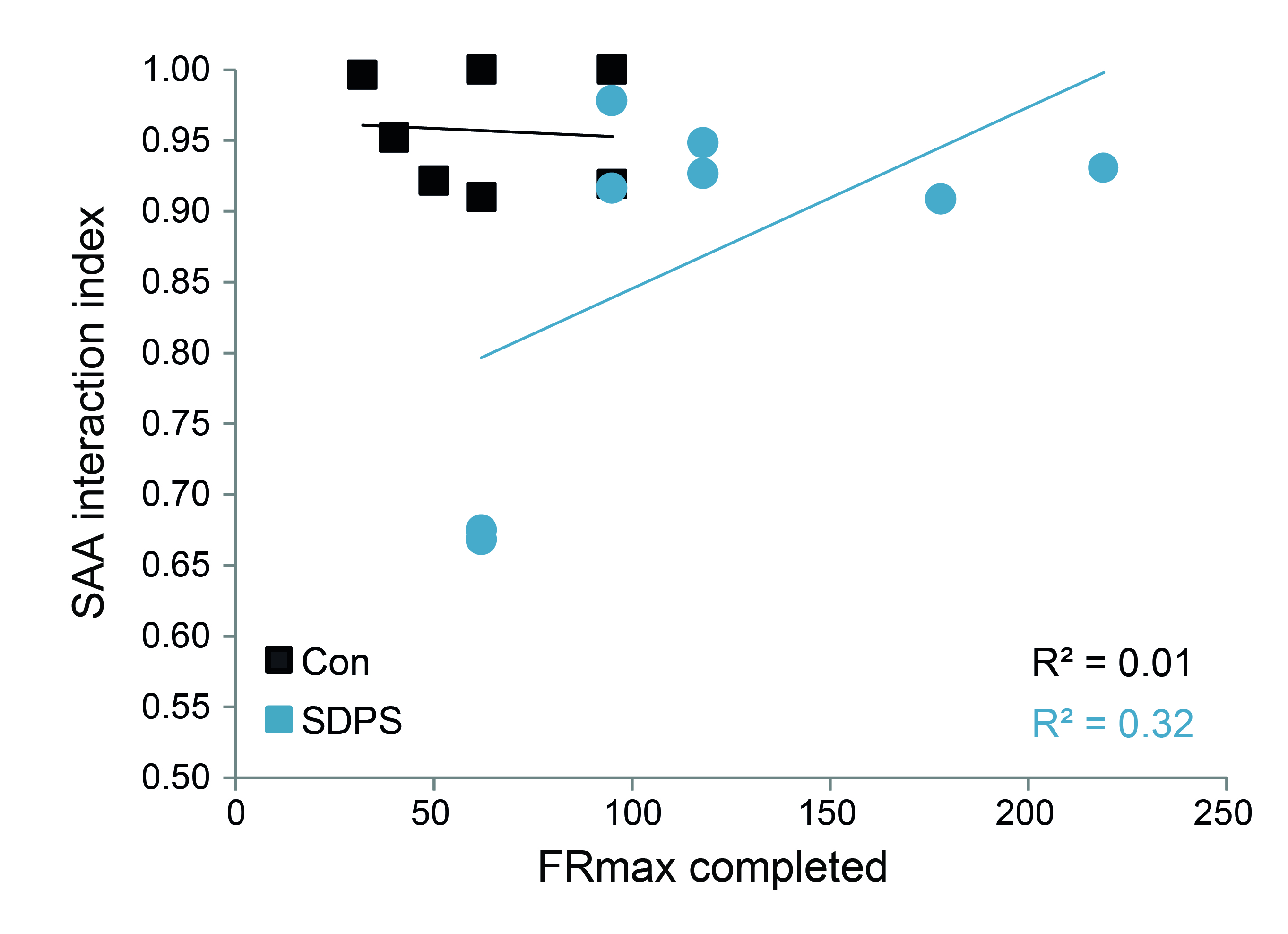


**Supp. Fig. 6. SDPS-induced social withdrawal does not predict motivation to seek sucrose.** The effect of SDPS in social behavior (SAA) was plotted against the breaking point (FRmax reached). Although SDPS avoidance behavior seemed to be associated with PR performance, no significant correlation was observed in either group: Con, *P*=0.87; SDPS, *P*=0.14.


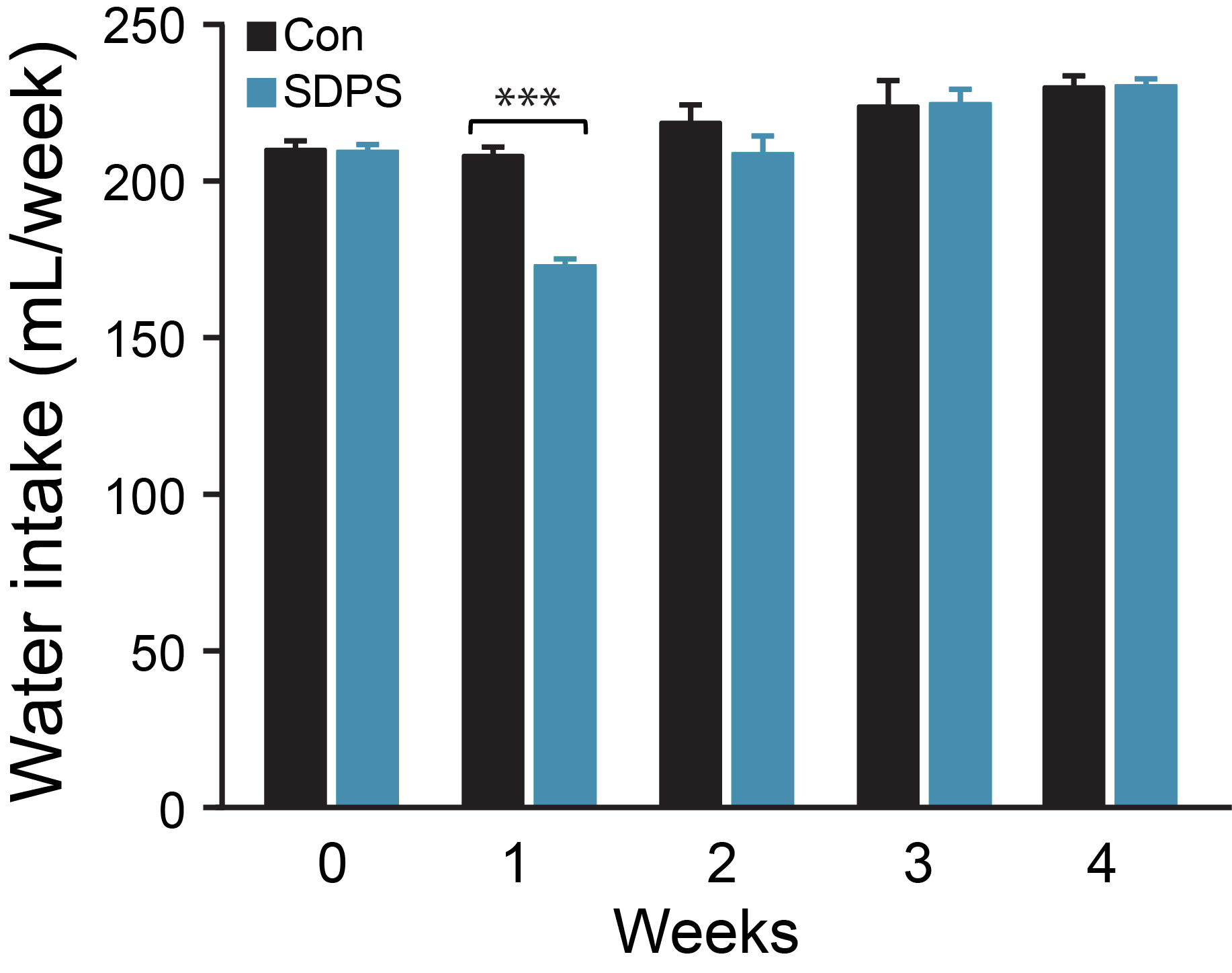


**Supp. Fig. 7.** **SDPS effects in home-cage water intake are transient.** In an independent set of animals, weekly water intake indicated that SDPS affects consumption only transiently, i.e., at the week following the defeat period. During the following weeks, water intake resumes to control levels, excluding differences in total fluid intake as a factor for the altered sucrose seeking behavior seen 2–3 months after SDPS. *** *P*<0.001.

**
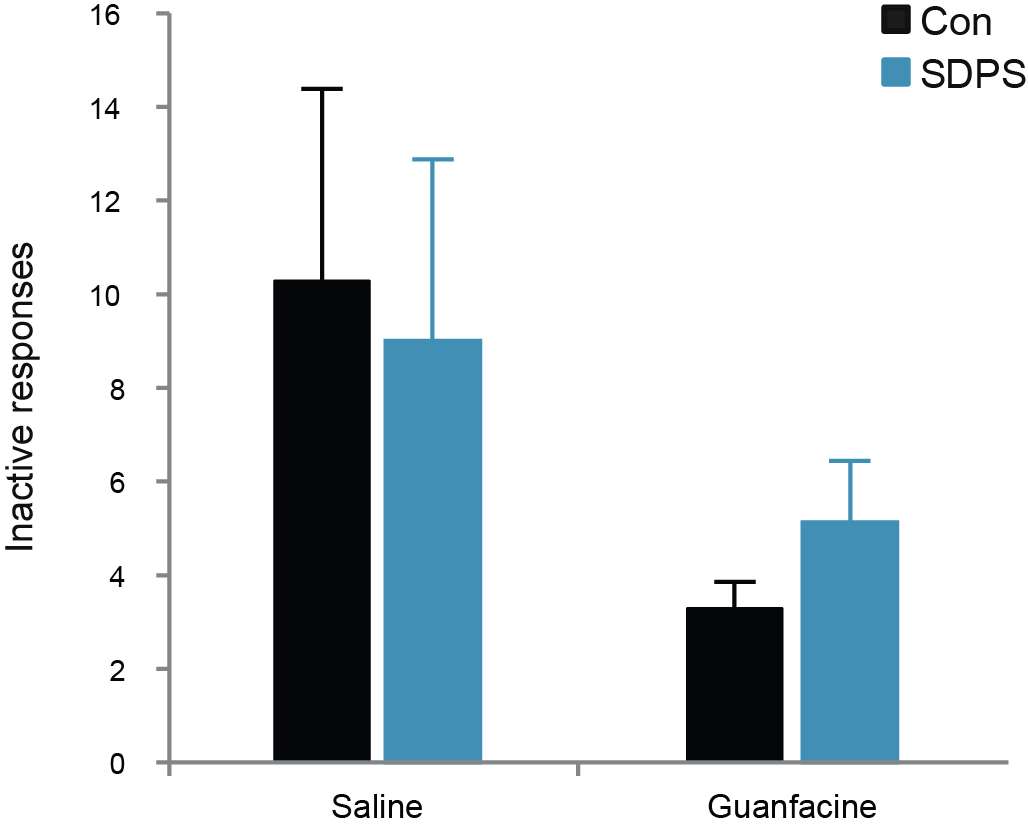
**

**Supp. Fig. 8.** **Guanfacine does not affect inactive responding during PR training.** Responding to the inactive hole remained low in both controls and SDPS animals during PR, and no between-group differences were observed in either saline (*P*=0.82) or guanfacine (*P*=0.24) sessions. Guanfacine lowered the number of responses to the inactive hole, but no statistically significant effects were detected (Con, *P*=0.12, SDPS, *P*=0.36), further supporting the lack of non-specific motoric effects.
